# Cisplatin treatment of testicular cancer patients introduces long-term changes in the epigenome

**DOI:** 10.1186/s13148-019-0764-4

**Published:** 2019-12-03

**Authors:** Cecilie Bucher-Johannessen, Christian M. Page, Trine B. Haugen, Marcin W. Wojewodzic, Sophie D. Fosså, Tom Grotmol, Hege S. Haugnes, Trine B. Rounge

**Affiliations:** 1 Department of Research, Cancer Registry of Norway, Oslo, Norway; 2 Oslo Centre for Biostatistics and Epidemiology, Section for Research Support, Oslo University Hospital, Oslo, Norway; 3 Centre for Fertility and Health, Norwegian Institute of Public Health, Oslo, Norway; 4 Faculty of Health Sciences, OsloMet - Oslo Metropolitan University, Oslo, Norway; 5 Department of Oncology, The Norwegian Radium Hospital/Oslo University Hospital, Oslo, Norway; 6 Faculty of Medicine, University of Oslo, Oslo, Norway; 7 Department of Oncology, University Hospital of North Norway, Tromsø, Norway; 8 Institute of Clinical Medicine, UIT The Arctic University of Norway, Tromsø, Norway; 9 Department of Informatics, University of Oslo, Oslo, Norway

**Keywords:** Cisplatin-based chemotherapy, Platinum, DNA methylation, Metabolic syndrome, Testicular cancer survivors, Epigenome-wide association study, Long-term effects, Epigenetic

## Abstract

**Background:**

Cisplatin-based chemotherapy (CBCT) is part of standard treatment of several cancers. In testicular cancer (TC) survivors, an increased risk of developing metabolic syndrome (MetS) is observed. In this epigenome-wide association study, we investigated if CBCT relates to epigenetic changes (DNA methylation) and if epigenetic changes render individuals susceptible for developing MetS later in life. We analyzed methylation profiles, using the MethylationEPIC BeadChip, in samples collected ~ 16 years after treatment from 279 Norwegian TC survivors with known MetS status. Among the CBCT treated (*n* = 176) and non-treated (*n* = 103), 61 and 34 developed MetS, respectively. We used two linear regression models to identify if (i) CBCT results in epigenetic changes and (ii) epigenetic changes play a role in development of MetS. Then we investigated if these changes in (i) and (ii) links to genes, functional networks, and pathways related to MetS symptoms.

**Results:**

We identified 35 sites that were differentially methylated when comparing CBCT treated and untreated TC survivors. The PTK6–RAS–MAPk pathway was significantly enriched with these sites and infers a gene network of 13 genes with *CACNA1D* (involved in insulin release) as a network hub*.* We found nominal MetS-associations and a functional gene network with *ABCG1* and *NCF2* as network hubs.

**Conclusion:**

Our results suggest that CBCT has long-term effects on the epigenome. We could not directly link the CBCT effects to the risk of developing MetS. Nevertheless, since we identified differential methylation occurring in genes associated with conditions pertaining to MetS, we hypothesize that epigenomic changes may also play a role in the development of MetS in TC survivors. Further studies are needed to validate this hypothesis.

## Background

After the introduction of cisplatin in the treatment of testicular cancer (TC) in the late 1970s [1], this malignancy has become a model for curative treatment even in case of metastatic disease. Cisplatin-based chemotherapy (CBCT) has been integrated into standard treatment of several cancers in addition to TC, including gynecological, lung, bladder, and head and neck cancer [2]. For men with metastatic TC, three to four cycles of cisplatin in combination with etoposide and bleomycin (BEP) comprise the cornerstone in the treatment of metastatic disease [3], yielding 5-year disease-specific survival rates > 90% [4].

Due to the excellent prognosis and young age at diagnosis, TC survivors can expect to live for 30–50 years after successful treatment [5]. However, the very long-term relative survival among TC survivors is lower than among the age-matched population [6], primarily related to increased risks of second cancers and cardiovascular disease (CVD) [5]. Metabolic syndrome (MetS) is a well described late effect after TC treatment and is a possible mediator of both the increased risk of second cancers as well as CVD [7–9]. The prevalence of MetS in the general population differs according to MetS definition and increases by age, affecting about 20–25% of most Western populations [10, 11]. MetS including hypertension, increased body mass index (BMI), pre-diabetic biochemical serum changes, and/or hyperlipidemia is a constellation of risk factors for CVD [12]. An increased age-adjusted odds ratio (OR) for developing MetS after CBCT has been found when compared with patients treated with only surgery [9, 13, 14], although these results are not quite consistent [15].

Cisplatin exposure has been shown to result in drug-induced DNA hypermethylation both in vitro and in vivo [16–19]. In recent years, evidence for epigenetic changes predisposing to MetS has also been documented [20–22]; it is therefore plausible that these changes caused by CBCT could be involved in the development of MetS. Identification of differential DNA methylation (DNAm) in TC survivors that develop MetS compared to those who do not could provide a better understanding for the underlying mechanisms behind this serious late effect.

We hypothesize that epigenetic changes caused by CBCT render TC survivors susceptible for developing MetS later in life. The aim of this study was to (i) evaluate the potential long-term effect of CBCT on the epigenome in a cohort of Norwegian TC survivors, and (ii) identify possible associations between epigenetic changes and development of MetS. We also investigated if these changes in (i) and (ii) links to genes, functional networks, and pathways related to MetS symptoms.

## Results

The basic characteristics of the TC survivors are outlined in Table 1. Median age at diagnosis ranged between 27 and 30 years, while median age at SII ranged between 47 and 51 years. Mean β methylation was 0.62 in all four groups.

**Table 1:** Characteristics at diagnosis and follow-up for patients (*N* = 279), split by treatment group. Median values and range are reported for the groups

	CBCT+^a^ MetS+^b^	CBCT− MetS+	CBCT+ MetS−	CBCT− MetS−	CBCT model *p* value	MetS model *p* value
N	61	34 (32^c^)	115	69		
Age at diagnosis (years)	30 (18–52)	30 (16–49)	27 (16–47)	28 (18–52)	0.09	< 0.001
Age at sample collection (years)	48 (29–64)	45 (28–74)	44 (23–61)	43 (26–62)	0.81	< 0.001
Age at survey II (years)	51 (36–69)	52 (36–68)	47 (31–66)	48 (33–68)	0.06	< 0.001
Time between surgery and sample collection (years)	17 (5–27)	18 (6–35)	17 (6–28)	14 (5–28)	0.02	0.02
Time between sample collection and MetS diagnosis (years)	0 (0–9)	8 (− 8–9)	0 (0–9)	8 (0–9)	<0.001	0.09
Clinical characteristics at diagnosis						
Initial disease stage					< 0.001	0.58
I	18	32	35	66		
IMK positive^d^/II	36	0	58	3^e^		
III	2	0	4	0		
IV	5	0	18	0		
Histology					0.16	0.15
Seminoma	8	1	6	2		
Non-seminoma	53	31	109	67		
Cumulative cisplatin dose (mg)	790 (570–920)		760 (495–1400)		< 0.001	
Selected characteristics at follow-up						
Testosterone (nmol/L)^f^	11.1 (3–26)	12 (3–24)	15 (3–32)	16 (6–38)	0.30	< 0.001
Physical activity					0.40	0.002
Very active	26	12	72	36		
Moderate	25	16	37	28		
Sedentary	10	4	5	4		
Smoking status					0.77	0.13
Never smoker (%)	43	33	51	49		
Former smoker (%)	34	42	24	28		
Current smoker (%)	23	24	24	23		
Mean β methylation	0.62	0.62	0.62	0.62	0.21	0.71

^a^*CBCT* cisplatin-based chemotherapy

^b^*MetS* metabolic syndrome

^c^N passed array filter quality

^d^*IMK* marker positive

^e^Rendered tumor free by surgery alone

^f^Regression analysis showed that testosterone level is (on average) 4.2 nmol/L lower in groups 1 and 2 (MetS+) compared with groups 3 and 4 (MetS−) when adjusted for age (*p* = 1.6 E-07)

### Study confounders

**Fig. 1:**
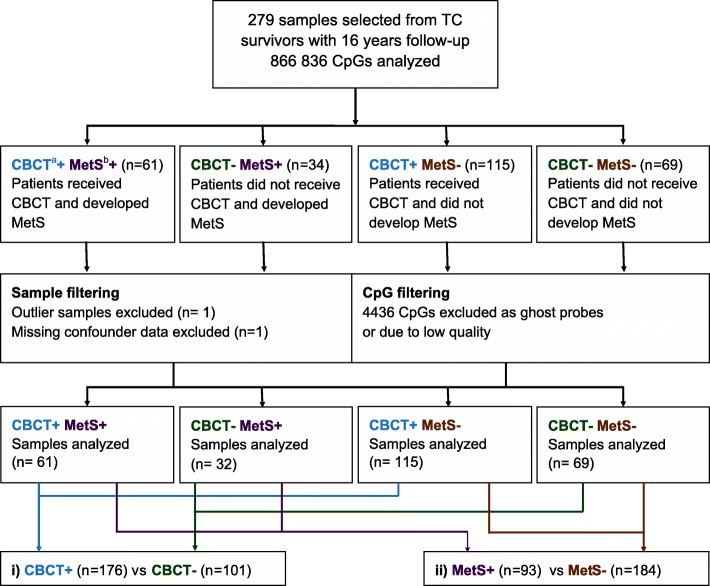
CONSORT flow diagram of included samples that were analyzed with the MethylationEPIC BeadChip (*n* = 279). Samples were from testicular cancer survivors divided into four groups according to CBCT and MetS status. ^a^ *CBCT* cisplatin-based chemotherapy. ^b^ *MetS* metabolic syndrome

Principal component analyses of the DNAm did not show differences between the four groups (Additional file 1: Figure S1). Global methylation was not associated with CBCT or MetS (*p* > 0.05) (Additional file 1: Figure S2). However, we identified associations between CBCT and relative proportions of CD4^+^ T cells (*p* = 0.0001), and CD8^+^ T cells (*p* = 0.04). Testosterone was also significantly associated with MetS (*p* = 1.6 E-07). We found 3109 Bonferroni significant CpGs (cytosine nucleotide followed by a guanine nucletide) (*p* value < 0.01) associated with age and 229 Bonferroni significant CpGs (*p* value < 0.01) associated with smoking habits, including two smoking related genes (Additional file 1: Figure S3) (Fig. 1)*.*

### Long-term effects of cisplatin on DNA methylation

**Fig. 2:**
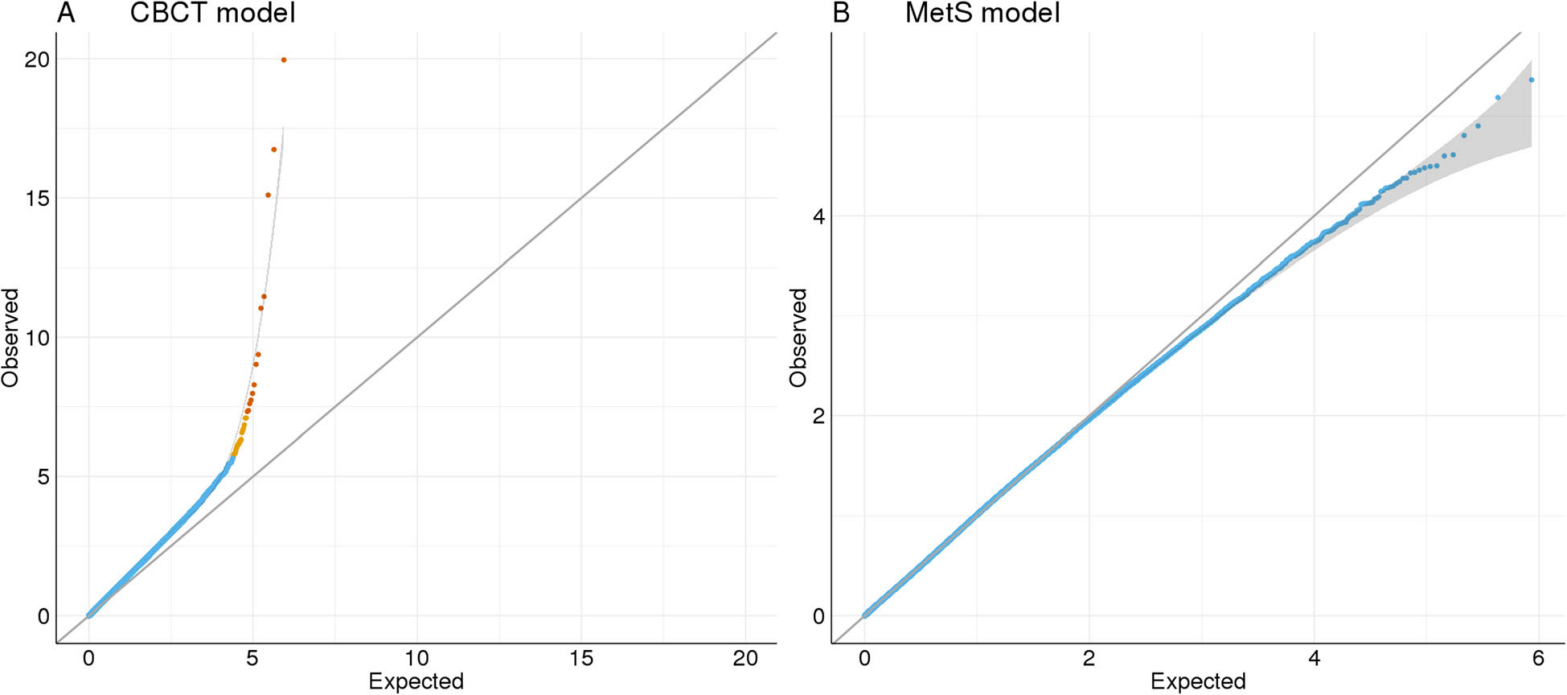
Q-Q plots for **a** cisplatin-based chemotherapy (CBCT) model, methylation β value as the dependent and CBCT as the independent variable, adjusted for smoking, age, testosterone, and cell count. **b** Metabolic syndrome (MetS) model, MetS as the dependent and methylation β value as the independent variable, adjusted for CBCT, smoking and age

**Fig. 3:**
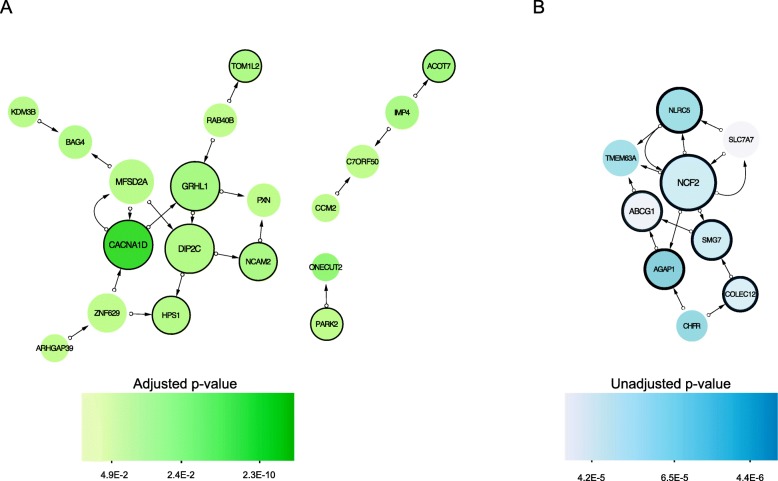
Functional gene networks of cisplatin-based chemotherapy (**a**) and metabolic syndrome (**b**) related genes reconstructed using the GENIUS tool. Nodes represent genes and edges (arrows) show the directions of the interactions found. Size of nodes is proportional to a gene network degree (number of neighbors of a given gene in the network). A node color intensity represents significance from the differential methylation analysis (high intensity colors represent highly significant genes, adjusted *p* value for panel (**a**) and unadjusted *p* value for panel (**b**) shown). Nodes marked with black circles represent genes associated with any of the metabolic syndrome trait

After adjusting for age, testosterone, smoking, and cell count, 35 CpG sites were associated with CBCT after False Discovery Rate (FDR) correction (Table 2). Of these, 13 CpG sites were significantly associated with CBCT after Bonferroni correction (Fig. 2a). Of the FDR-associated CpGs, 21 were located in Open Sea, three in a CpG island, and 11 in either CpG island shelf or shore. The different multivariate models showed similar results (Additional file 1: Figure S4B and Figure S5B). Nineteen annotated CBCT genes were found in the GENIUS database. One network-structure was identified for 13 genes, of which six were related to MetS. Each node had an average of 2.0 neighbors. *CACNA1D*, *DIP2C*, and *GRHL1* had the highest network degrees and were all associated with MetS (Fig. 3a).

**Table 2:** Annotations for the 35 FDR significant CpGs (adjusted *p* values < 0.05) for cisplatin-based chemotherapy (CBCT) model

CpG name	Gene name^a^	FDR^b^	Bonf^c^	Function of the gene product	Disease/Trait association^d^
cg07677157	*RP11*-*221N13.4*	9.56E-15	9.56E-15		
cg08057120		7.78E-12	1.56E-11		
cg26408927	*CACNA1D*	2.26E-10	6.79E-10	Mediate the entry of calcium ions into excitable cells. Involved in a variety of calcium-dependent processes. Regulates intracellular processes such as contraction, secretion, neurotransmission and other gene expression	Sinoatrial node dysfunction and deafness. Hypertension. Body mass index. Insulin resistance/response. Systolic blood pressure. Diastolic blood pressure. Visceral adipose tissue/subcutaneous adipose tissue ratio. Type 2 diabetes
cg27487222		7.37E-07	2.95E-06		
cg11031221	*LINC00511*	1.56E-06	7.78E-06	A long non-protein coding RNA, involved in the regulation of gene expression during tumor progression	
cg22688137		6.01E-05	< 0.001		
cg24833462	*AC023672.2*	< 0.001	< 0.001		
cg20063141	*ONECUT2/* *AC090340.1*	< 0.001	0.004	This gene encodes a member of the one cut family of transcription factors, which are characterized by a cut domain and an atypical homeodomain	
cg08889373	*ACOT7/RP1*-*202O8.3*	0.001	0.009	Protein hydrolyzes the CoA thioester of palmitoyl-CoA and other long-chain fatty acids	Type 2 diabetes. Atherosclerosis
cg14792781	*GRHL1*	0.002	0.016	Is a transcription factor necessary during development	Cancer. Type 1 diabetes
cg14634473		0.002	0.021		
cg21940081	*IMP4*	0.003	0.037	Part of the 60-80S U3 small nucleolar ribonucleoprotein (U3 snoRNP) complex. Responsible for early cleavage steps of pre-18S ribosomal RNA processing	
cg03877706	*NCAM2*	0.003	0.041	Belongs to the immunoglobulin superfamily. May play important roles in selective fasciculation and zone-to-zone projection of the primary olfactory axons	Obesity. Visceral fat
cg00303773	*TOM1L2*	0.005	0.070	Participate in vesicular trafficking. Play a role in endosomal sorting	Body mass. Type 2 diabetes
cg10113471		0.005	0.069		
cg23304747	*PITPNM2*	0.008	0.123	Catalyzes the transfer of phosphatidylinositol and phosphatidylcholine between membranes (in vitro). Binds calcium ions	
cg14972510	*BAG4*	0.010	0.167	Inhibits the chaperone activity of HSP70/HSC70. Prevents constitutive TNFRSF1A signaling. Negative regulator of PRKN translocation to damaged mitochondria	
cg26561082	*DIP2C*	0.011	0.197	The protein shares strong similarity with a Drosophila protein which interacts with the transcription factor disco and is expressed in the nervous system	Blood metabolite levels
cg24869056	*HPS1*	0.012	0.235	Play a role in organelle biogenesis associated with melanosomes, platelet dense granules and lysosomes	Obesity-related traits
cg14629524	*KDM3B*	0.020	0.401	Histone demethylase that specifically demethylates Lys-9 of histone H3, thereby playing a central role in histone code	
cg04156896	*MFSD2A*	0.021	0.459	Transmembrane protein and sodium-dependent lysophosphatidylcholine transporter involved in the establishment of the blood-brain barrier	
cg27367992	*ST6GAL1*	0.021	0.451	Catalyzes the transfer of sialic acid from CMP-sialic acid to galactose-containing substrates	Type 2 diabetes
cg08343240	*AC008703.1*	0.024	0.573	RNA gene	
cg27545041		0.024	0.567	An important paralog of this gene is INTS6 / RNA Gene and is affiliated with the non-coding RNA class	
cg04046944	*CACNA1S*	0.025	0.614	This gene encodes one of the five subunits of the slowly inactivating L-type voltage-dependent calcium channel in skeletal muscle cells	
cg06225648		0.025	0.651		
cg12381697	*CCM2*	0.028	0.751		
cg13207339	*PARK2*	0.030	0.849	A component of a multiprotein E3 ubiquitin ligase complex that mediates the targeting of substrate proteins for proteasomal degradation	Metabolite levels. Body mass index. Aging
cg22345432	*PXN/* *PXN-AS1*	0.033	0.972	Involved in actin-membrane attachment at sites of cell adhesion to the extracellular matrix (focal adhesion)/RNA Gene and is affiliated with the non-coding RNA class	
cg17158941	*C7orf50*/*AC073957.15*	0.040	1	Chromosome 7 Open Reading Frame 50	C-reactive protein levels or total cholesterol levels
cg03289031	*ZNF629*	0.040	1		
cg26540402	*PRF1*	0.040	1	Plays a key role in secretory granule-dependent cell death and in defense against virus infected or neoplastic cells	
cg16657582		0.043	1		
cg21902759	*RAB40B*	0.043	1	Substrate-recognition component of a SCF-like ECS (Elongin-Cullin-SOCS-box protein) E3 ubiquitin ligase complex which mediates the ubiquitination and subsequent proteasomal degradation of target proteins	
cg19377056	*ARHGAP39*	0.049	1		

^a^UCSC gene name

^b^FDR - CBCT False Discovery Rate significance (*p* < 0.05)

^c^Bonf - CBCT Bonferroni significance (*p* < 0.05)

^d^Selected from GeneToFunction database (human only) and Gene Cards disease associations

### DNA methylation and risk of developing MetS

We could not identify MetS differentially methylated CpG sites after adjusting for age, smoking, CBCT, and multiple testing (FDR or Bonferroni) (Fig. 2b). We present the top 15 differentially methylated CpG sites (unadjusted *p* values) (Table 3) of which 11 CpGs were located on an open sea and four on the CpG island shores. None of the multivariate models showed epigenome-wide association study (EWAS) significant results, and their top hits differed (Additional file 1: Figure S5B). In addition, models for the individual MetS components (hypertension, cholesterol, waist circumference, fasting glucose, and triglycerides) did not give EWAS significant associations. There was no overlap between the top 2000 nominally significant CpGs for these five MetS component models and the MetS model (Additional file 1: Figure S6).

**Table 3:** The 15 CpG sites with lowest unadjusted *p* values for associations between DNA methylation (DNAm) and metabolic syndrome (MetS)

CpG name	Gene^a^	P^b^	Gene function	Disease/Trait association^c^
cg01562302	*SLC7A7*	4.37E-06	Involved in the sodium-independent uptake of dibasic amino acids and sodium-dependent uptake of some neutral amino acids	
cg06500161	*ABCG1*	6.50E-06	Involved in macrophage cholesterol and phospholipids transport, and may regulate cellular lipid homeostasis in other cell types	Type 1 diabetes
cg05489343	*COLEC12*	1.26E-05	Scavenger receptor associated with host defense, C-lectin family, proteins that possess collagen-like sequences and carbohydrate recognition domains	Obesity-related traits
cg07203167	*NCF2/SMG7*	1.57E-05	Required for activation of the latent NADPH oxidase	Insulin resistance
cg23064281		2.44E-05		
cg22084453		2.51E-05		
cg09209794	*TMEM63A*	3.15E-05	Acts as an osmosensitive calcium-permeable cation channel	
cg23167087	*TTC18*/*CFAP70*	3.20E-05		
cg16007266	*NLRC5*	3.31E-05	Plays a role in cytokine response and antiviral immunity through its inhibition of NF-kappa-B activation and negative regulation of type I interferon signaling pathways	HDL cholesterol
cg14810357	*AC064875.2*	3.49E-05		
cg09120938	*CHFR*	3.67E-05	Regulates cell cycle entry into mitosis and, therefore, may play a key role in cell cycle progression and tumorigenesis, belongs to DNA damage pathway	
cg02255098	*BCAM*	3.71E-05	A receptor for the extracellular matrix protein, laminin.	Waist-to-hip circumference ratio
cg22926824	*AGAP1*	4.18E-05	Direct regulator of the adaptor-related protein complex 3 on endosomes	Cardiovascular disease in hypertension (calcium channel blocker interaction)
cg22003124		4.21E-05		
cg16307144	*DPF1*	4.55E-05	Gene Ontology (GO) annotations related to this gene include nucleic acid binding	

^a^Gene UCSC gene name

^b^*P* value unadjusted *p* value

^c^Selected from GeneToFunction database (human only) and Gene Cards disease associations

We identified one network-structure for the nine MetS-associated genes found in the GENUS database (Fig. 3b).

### Overlap between CBCT and MetS associations

There were no FDR or Bonferroni significantly differentially methylated CpG sites associated with both CBCT and MetS. However, the comparison of the top 2000 CpG sites associated with CBCT and MetS with unadjusted *p* values < 0.05 for both analyses identified ten common CpG sites (Table 4).

**Table 4:** Annotations for the nine overlapping CpGs for the cisplatin-based chemotherapy (CBCT) model and metabolic syndrome (MetS) model for the 2000 CpGs with the lowest unadjusted *p* values < 0.05

Cpg name	Gene^a^	P^b^ CBCT model	P^b^ MetS model	Gene function	Disease/Trait association^d^
cg25165017		< 0.001	0.001		
cg27087650	*BCL3*	< 0.001	0.012	Contributes to the regulation of cell proliferation	
cg10785263		< 0.001	0.020		
cg17986793	*MX1*	< 0.001	0.022	Gene product, Interferon-induced GTP-binding protein Mx1 is a protein that in humans is encoded by the MX1 gene	
cg10587886	*LMCD1-AS1*	< 0.001	0.044	LMCD1 antisense RNA 1	
cg18871648	*ELMSAN1*	< 0.001	0.050	ELM2 and Myb/SANT domain containing 1	
cg07688244		< 0.001	0.098		
cg14792781	*GRHL1*	< 0.001	0.127	This gene encodes a member of the grainyhead family of transcription factors. The encoded protein can exist as a homodimer or can form heterodimers with sister-of-mammalian grainyhead or brother-of-mammalian grainyhead. This protein functions as a transcription factor during development	
cg25273039	*NXPH1*	< 0.001	0.165	The product protein forms a very tight complex with alpha neurexins, a group of proteins that promote adhesion between dendrites and axons	Waist-to-hip ratio adjusted for body mass index, Obesity-related traits
cg19509829	*ATP2A2*	< 0.001	0.215	Product Belongs to a family of ATPase enzymes that helps control the level of positively charged calcium atoms (calcium ions) inside cells	Glucose homeostasis traits

^a^Gene UCSC gene name

^b^*P* unadjusted *p* value

^c^Selected from GeneToFunction database (human only) and Gene Cards disease associations

### Pathway enrichment for CBCT-associated CpGs

Genes in approximation to 78 differentially methylated CpG sites (FDR < 0.1) associated with CBCT were analyzed for gene enrichment to provide a functional interpretation of our results. We identified the “*PTK6 Regulates RHO GTPases*, *RAS GTPase*, and *MAP kinase*” Reactome pathway as significantly enriched (adjusted *p* value = 0.03). For GO biological process, we found the “cellular response to growth hormone stimulus” pathway significantly enriched (*p* value = 0.005). For GO cellular component, the “L-type voltage-gated calcium channel complex” pathway was significantly enriched (*p* value = 0.02). We did not find significant pathways for KEGG and GO molecular function.

### Differentially methylated regions associated with CBCT and MetS

Using bumphunting, 419 regions (bumps) were identified; however, none were significantly associated with CBCT based on adjusted *p* values (data not presented). Neither did we identify significant hits when using DMRcate for the two model (data not presented).

We checked if genomic coordinates of the CpGs of interest were present as SNPs in GWAS Central database. We did not find any of the CBCT, MetS, and overlapping CpG sites from Tables 2, 3, and 4, respectively. In the EWAS Atlas database, we found one association with post-obese (cg07677157), and one association with high-saturated fatty acids diet (cg07677157) for CBCT-associated CpGs. From our CpG sites related to MetS, there was one hit cg06500161 (ABCG1), associated with MetS, BMI, and type 2 diabetes in this database. In the overlapping CpGs, we found cg27087650 associated with BMI.

## Discussion

In this EWAS, we identified lasting CBCT-related effects in 35 differentially methylated CpG sites across the genome, on average 16 years after treatment. These effects may be attributed to the initial CBCT, and/or to the small amount of platinum persistent in serum post-treatment [5]. We found insulin- and body mass-related genes in proximity to the CBCT-associated hits, supporting our hypothesis that the CBCT-MetS relationship is linked to epigenetics. Interestingly, we found CpG sites in proximity to the gene *ABCG1*, which has been associated to body mass, triglycerides, HDL-C, atherosclerosis, and type 2 diabetes in EWAS [23–25], among our nominally significant MetS CpGs.

The gene closest to the CBCT− top hit, cg07677157, is *RPSAP52*, a gene linked to type 2 diabetes in genome-wide association studies (GWAS) [26]. The top annotated CBCT gene, *CACNA1D*, encodes voltage-dependent calcium channels, which regulate insulin release. Polymorphisms in *CACNA1D* are also associated with type 2 diabetes [27], linked to diastolic and systolic blood pressure [28], and ototoxicity [29]. Other CBCT-associated genes were *ACOT7*, implicated in the pathophysiology of type 2 diabetes [30] and atherosclerosis [31]; *GRHL1*, encoding a transcription factor involved in epithelial development and linked to several types of cancer, cardiovascular diseases, and type 1 diabetes; and *TOM1L2*, linked to body mass and type 2 diabetes in GWAS [32] (Table 2).

We found “PTK6 Regulates RHO GTPases, RAS GTPase, and MAP kinases” which is part of the RAS signal transduction pathway enriched with CBCT-associated methylation. The pathway regulates cell differentiation and plays a role in cell proliferation [33–35], which might be a relevant mechanism in relation to the increased risk of second cancer after CBCT in TC survivors [36]. The pathway “cellular response to growth hormone stimulus” is also enriched for CpGs associated to CBCT. The involvement of these oncogenic pathways is consistent with the cytotoxicity of CBCT, which is interesting considering the rather long time window between treatment and DNAm measurements.

Examples of MetS-associated genes include *COLEC12*, encoding a scavenger receptor involved in several functions associated with host defense; *NCF2*, for which increased expression has been observed in patients with insulin resistance [37]; and *SMG7*, playing a role in p53 function in response to DNA damage [38].

The possible relationship between CBCT-induced DNAm and susceptibility for developing MetS was analyzed by exploring the sequence of events separately, as there are no well-established tools for doing high-dimension mediation analysis that fit with our design. The overlap between the two models, including the top 2000 hits, was ten sites (Table 4). These might be spurious findings since associations were nominal. The small overlap might suggest two unrelated mechanisms leading to MetS which was supported by publicly available distinct EWAS (EWAS Atlas). Nonetheless, for both models, the majority of top CpGs was linked to factors pertaining to MetS. Additionally, among the genes that clustered in the network analysis, those with the highest network degree (*CACNA1D*, *DIP2C*, and *GRHL1* for CBCT, and *ABCG1* and *NCF2* for MetS) were all associated with MetS. The network analyses suggest that CBCT and MetS-related effects may be mediated synergistically. We speculate that changes in methylation in these clustered genes might affect gene expression, thus increasing the likelihood of developing MetS. These findings support the notion that DNAm may mediate the effect of CBCT on MetS risk later in life. Validation and replication of the top results are needed in an independent cohort. Inclusion of a non-oncological control group, with and without MetS, will further elucidate if the findings are independent of tumor intrinsic factors, and if the MetS hits are related to CBCT.

The survivors in our study who developed MetS have somewhat lower testosterone levels than those without MetS, regardless of CBCT, thus low testosterone may have confounded the results. Previous studies have shown that lower total testosterone level is associated with higher risk of developing MetS [39]. Independently of the model used, testosterone did not markedly alter the significant associations. This indicates that the MetS DNAm association is not attributable to low testosterone levels.

Strengths of the study include a reliable and broadly characterized study population which has been followed for many years. In addition, we have considered the most important confounding factors by matching the groups. Furthermore, the epigenetic analyses have been performed on EPIC BeadChip arrays, which provides state-of-the art tool for epigenome-wide association analyses, covering over 850,000 CpG sites. The EPIC array represents a significant improvement compared to its predecessor, the HM450 array, with increased genome coverage of regulatory regions [40]. Finally, we used curated annotation resources and updated GRCh38/hg38 genome [41].

Some limitations of the present study should also be considered. Even though the total number of TC survivors was large, the sample size of those treated with surgery only and developed MetS was 34. Due to the limited sample size, we chose the minimally adjusted model for MetS. A power issue may thus contribute as to why there were no differentially DNA methylated CpG sites associated with MetS after adjusting for multiple testing. We were not able to distinguish between the effect of the initial CBCT and the effect of platinum residuals [5] as this would have needed a time-series analysis. It is not possible to rule out that some of the differential DNA methylation associated to CBCT could be related to tumor-specific intrinsic factors. However, TC stage markers of DNA methylation including HOXA9, RASSF1A, and SCGB3A1 [42] were not observed, indicating that potential confounding by such factors was unlikely. The possible impact of second primary cancer is negligible due to long time span between sampling and diagnosis.

## Conclusions

Our results suggest that CBCT has long-term effects on the epigenome. Although we could not directly link the CBCT effects to the risk of developing MetS, it may still play a role in the development of MetS in TC survivors. This is supported by the observation that the differential DNAm occurs in genes related to MetS. Furthermore, our results contribute to a better understanding of the cellular mechanisms behind the development of MetS in TC survivors treated with CBCT. Although the influence of CBCT on the epigenome is plausible, validation of the observed differential methylation of specific CpGs is necessary. Our findings also indicate that other adverse effects of CBCT, such as ototoxicity, may be mediated by epigenetic changes. These topics could be subject to future studies, also encompassing other cancer forms using CBCT, and risk of second cancer. In terms of clinical perspective, our results may provide early identification of individuals with increased risk for development of MetS.

## Methods

### Study population and patient assessments

Participants were long-term survivors of unilateral TC diagnosed from 18 to 75 years of age, treated between 1980 and 1994. The original national cohort consisted of 1463 men (Caucasians) who participated in follow-up survey I (SI) at five Norwegian university hospitals during the period 1998–2002. In total, 990 males, younger than 60 years old at SI, were subsequently evaluated with regard to CVD and MetS in a second survey (SII) performed 2007–2008 [15]. A third survey (SIII) was performed in 2015–2016. Overall, 279 participants with MetS data obtained in SII, and frozen whole blood samples from either SI (*n* = 137), SII (*n* = 132), or SIII (*n* = 8) available for DNA analyses, were included in the present study. We included the samples that best fitted the matching criteria (see below). For those who had samples from more than one survey, we chose the DNA obtained at the earliest time point after diagnosis to capture as much of the CBCT related effects as possible.

Data from questionnaires, clinical examinations (including blood pressure and waist circumference measurements), and laboratory tests (including fasting blood glucose and blood lipid measurements) were retrieved from SII. Smoking status was classified from questionnaire data into three groups: never, former, and current smoker. Age was used as a continuous variable in all statistical analyses. All routine blood samples were analyzed at the Oslo University Hospital. Plasma levels of lipids and glucose were measured enzymatically. Serum levels of testosterone were determined using immunoassays. MetS was defined according to the National Cholesterol Education Program expert panel, as the presence of minimum three of the following five criteria: blood pressure ≥ 130/85 mmHg, HDL-cholesterol < 1.0 mmol/L, triglycerides ≥ 1.7 mmol/L, waist circumference > 102 cm, and fasting glucose ≥ 6.5 mmol/L [43, 44]. Epigenomic changes related to MetS were assumed to be present at the sampling time point. Data regarding initial tumor stage, histology, and treatment details were retrieved from medical records. Treatment details according to stage and histology [45], CVD risk, and morbidity data from SI and SII have been published previously [9, 15, 46, 47]. Cancer Registry follow-up showed that 51 survivors acquired a second primary cancer within the cohort follow-up time (SIII). These cancers were diagnosed on average 6 (SD = 3) years after sample donation.

From the 279 TC survivors included, 103 had surgery only (orchiectomy with or without retroperitoneal lymph node dissection for selected cases) due to stage I disease, whereas 176 had undergone both surgery and CBCT (all with metastatic disease) (Fig. 1). From the surgery only (CBCT−) and the treatment (CBCT+) groups, we selected similar fractions of patients with MetS. These four groups were frequency-matched on smoking habits and age at blood sampling, allowing no more than two years difference in mean age. However, complete matching was not possible due to limited number of patients categorized as CBCT+ and MetS−. From this 2 × 2 design, we analyzed the data using a CBCT model and a MetS model, where all cases were included in both models (Fig. 1). Comparing the top hits from the two models enables the identification of CpG sites associated with both CBCT and MetS. This study was approved by Regional Ethical Committee (REC) south east D, reference 2015/1332.

### DNA methylation profiling

Genomic DNA was isolated from whole blood using standard chloroform–phenol extraction method. DNA concentration and purity of the DNA samples were analyzed using a NanoDrop ND-1000 (Thermo Fisher Scientific, Waltham, MA, USA). DNA from ten samples was isolated using QIAamp DNA Blood Mini Kit (Qiagen) and the Qiacube (Qiagen) according to manufacturer’s protocol. Of the 279 samples, four samples had only 300 ng (500 ng recommended); however, they showed good array quality in all control steps. Bisulfite conversion of the genomic DNA was done using the EZ DNA Methylation Kit (Zymo) and whole-genome DNAm were analyzed with the Infinium MethylationEPIC Kit (Illumina) according to manufacturer’s recommendations. This array covers 850,000 individual CpGs at CpG islands, RefSeq genes, ENCODE open chromatin, ENCODE transcription factor binding sites, and FANTOM5 enhancers sites. The 279 samples were randomized on three 96-well plates according to the four groups described.

The resulting raw data were analyzed using minfi v.1.20.2 in the R statistical environment v3.3.3 [48]. For details, see Supplementary method information. Two samples were excluded, one due to missing smoking information, and one being an outlier in the principal component analyses (Additional file 1: Figure S1). This resulted in a quality-controlled dataset of 277 samples and 862,400 CpG sites. CpG sites were mapped to the human genome (GRCh37/hg19) using the annotation file provided by the manufacturer (Illumina) [49] and further curated and translated to GRCh38/hg38 [41]. Additional information were retrieved from the UCSC genome browser [50, 51] and GeneCards (www.genecards.org) [52] and Gene2Function (http://www.gene2function.org) [53]. Relative proportion of cell types (B cells, CD4^+^ T cells, CD8^+^ T cells, natural killer cells, granulocytes, and monocytes) from the methylation profiles were estimated using the reference-based Houseman method [54, 55].

We deployed GENIUS (GEne Networks Inference Using Signatures) tool to predict local gene networks and key genes for biological functions [56]. The network was constructed using supervised machine learning method to find expression signatures. Input for the gene network was the FDR significant genes from the CBCT and MetS model. The network was visualized with Cytoscope 3.7.1 according to the nodes connectivity and degree [57].

### Statistics

The methylation values were transformed to β values (between 0 and 1), representing the intensity of methylation [58]. We used a linear regression model to investigate if cell type composition was associated with CBCT, adjusting for age at blood sampling and smoking habits. We also tested if MetS was associated with testosterone levels, adjusting for age. The results indicated that testosterone and cell type (five out of six cell types, B cells was dropped) composition might be confounders. The robust linear CBCT model with DNA methylation status as the dependent variable was therefore adjusted for age, smoking, cell type, and testosterone. To investigate the associations between DNAm and MetS with the latter as the dependent variable, we used a generalized logistic regression model. The MetS model was adjusted for age, smoking, and CBCT. Models with the best fit were included, and additional regression models tested are available in the Supplementary information (Additional file 1: Figure S4 and Figure S5). Additionally, separate generalized logistic regression models with the five variables underlying the MetS diagnosis (hypertension, cholesterol, waist circumference, fasting glucose, and triglycerides) as dependent variable and with the same covariates were tested. To adjust for multiple testing, Benjamini and Hochberg’s FDR [59] and Bonferroni correction [60] were applied to all models. Differentially methylated CpG sites, identified in the CBCT model and the MetS model, were defined as the intersection of the top 2000 hits with the lowest unadjusted *p* values.

In order to identify differentially methylated regions (DMR), we used two approaches, bumphunter [61] and dmrcate [62–64]. Bumphunter (v.1.20.0) was run with 1000 permutations and the cutoff was set to 0.05, corresponding to 5% difference on the β values on the CBCT model. We ran the DMRcate function (v.1.14.0) with default settings (max gap 1000 nucleotides between two significant probes and DNAm as outcome) on the CBCT model and by flipping the function around with the dichotomous variable as outcome on the CBCT and MetS models.

### Gene enrichment analysis and CpG characterization

CpG sites passing an FDR adjustment (*p* value < 0.1) were used to assess pathways enriched for differential DNAm. We employed Enrichr (http://amp.pharm.mssm.edu/Enrichr) [65, 66] a platform for KEGG [67], Reactome 2016 (v.62), and Gene Ontology (GO) 2018 (biological process, molecular function, and cellular component) pathways analysis [68, 69]. We performed an unweighted analysis, and reported *p* values are based on Fisher’s exact test.

We employed GWAS Central database (www.gwascentral.org) to evaluate if any of CpG sites of interest were previously reported as known SNPs [70]. We further scanned for associations between CpG sites of interest and known epigenome-wide associations from literature studies. We used the EWAS Atlas resource (https://bigd.big.ac.cn/ewas/index) [71]. This database features a large number of high-quality, manually curated, EWAS associations.

## Supplementary information

**Additional file 1: Figure S1.** PCA plot of β-methylation for the four sample groups, showing one outlier. The plot is coloured by different groups. Blue group did not develop MetS, but received CBCT, green did not develop MetS and did not receive CBCT, red developed MetS and received CBCT, and black developed MetS and did not receive CBCT. **Figure S2.** Barplot of the global average methylation per sample. Blue and green indicate whether patients had received cisplatin or not, respectively. Samples were sorted descending using their average methylation value. **Figure S3.** Boxplot of smoking associated CpGs for the genes *AHRR* and *F2RL3*. Never, Former and Current, refer to the smoking status as presented in Table 1. **Figure S4.** Q-Q plots for A) CBCT model, methylation β-value as the dependent and CBCT as the independent variable, adjusted for smoking, age, and cell count. B) MetS model, MetS as the dependent and methylation β-value as the independent variable, adjusted for CBCT, smoking, age and cell count. **Figure S5.** Q-Q plots for A) CBCT model, methylation β-value as the dependent and CBCT as the independent variable, adjusted for smoking, and age. B) MetS model, MetS as the dependent and methylation β-value as the independent variable, adjusted for CBCT, smoking, age, testosterone and cell count. **Figure S6.** Venn-diagram illustrating the overlapping number of top 2000 nominally significant CpGs between the original model, and the models with the 5 individual criteria of the MetS-diagnosis as dependent variable. Criteria is according to the National Cholesterol Education Program expert panel: Hypertension = blood pressure ≥130/85 mmHg, HDL = HDL-cholesterol <1.0 mmol/L, Triglycerides = triglycerides ≥1.7 mmol/L, Waist Circ. = waist circumference >102 cm, and Glucose = fasting glucose ≥6.5 mmol/L.

## Data Availability

The datasets generated and analyzed during the current study are not publicly available since individual privacy could be compromised, but are available from the corresponding author on request and with appropriate approvals.

## Abbreviations

BEP: Bleomycin, etoposide, and platinum
BMI: Body mass index
CBCT: Cisplatin-based chemotherapy
CpG: Cytosine nucleotide followed by a guanine nucleotide
CVD: Cardiovascular disease
DMR: Differentially methylated regions
DNAm: DNA methylation
MetS: Metabolic syndrome
TC: Testicular cancer
