# Supplementary material for: Cisplatin treatment of testicular cancer patients introduces long-term changes in the epigenome
Source: Clin Epigenetics. 2019 Dec 3;11:179. doi: 10.1186/s13148-019-0764-4 (PMC6892132; doi:10.1186/s13148-019-0764-4)

Additional file 1: **Cisplatin treatment of testicular cancer patients introduces long-term changes in the epigenome**

Cecilie Bucher-Johannessen[1], Christian M. Page[2,3], Trine B. Haugen[4], Marcin W. Wojewodzic[1], Sophie D. Fosså[1,5,6], Tom Grotmol[1], Hege S. Haugnes[7,8#] Trine B. Rounge[1,9#]

[#] equal contribution

Supplementary method information:

Processing of raw array data: Background subtraction and control normalization were done using preprocessIllumina in minfi package. Further normalization was done with the algorithm BMIQ from the wateRmelon package (v.1.22.1), which accounts for differences in probe type [1]. Quality control steps included checking for: 1) technical variation and outliers, 2) bias related to plate and well number, and 3) distribution in global methylation between the groups. No row or plate bias were observed across plates. Probes with no value or low detection quality were removed. One sample was excluded after filtering for missing smoking information, and one was excluded as an outlier in the principal component analyses (Figure S1).

In silico cell type composition prediction: The implemented "estimateCellCounts" function in the minfi package [2] were used for estimated cell type composition. We estimated cell type proportion of the samples which included Natural Killer cells, Granulocytes, CD8 T-cells (CD8T), CD4 T-cells (CD4T), Monocytes and Beta cells [2, 3]

Covariates in regression models: In addition to the covariates presented in the main text we also tested the following models:

- CBCT model - age, smoking (Fig. S5A)
- CBCT model - age, smoking, and cell type (Fig. S4A)
- MetS model - age, smoking, CBCT and cell type (Fig. S4B)
- MetS model - age, smoking, CBCT, cell type and testosterone (Fig. S5B)

References:

1. Teschendorff AE, Marabita F, Lechner M, Bartlett T, Tegner J, Gomez-Cabrero D, Beck S: **A beta-mixture quantile normalization method for correcting probe design bias in Illumina Infinium 450 k DNA methylation data.** *Bioinformatics* 2013, **29:**189-196.
2. Houseman EA, Accomando WP, Koestler DC, Christensen BC, Marsit CJ, Nelson HH, Wiencke JK, Kelsey KT: **DNA methylation arrays as surrogate measures of cell mixture distribution.** *BMC bioinformatics* 2012, **13:**86.

3. Reinius LE, Acevedo N, Joerink M, Pershagen G, Dahlen SE, Greco D, Soderhall C, Scheynius A, Kere J: **Differential DNA methylation in purified human blood cells: implications for cell lineage and studies on disease susceptibility.** *PLoS One* 2012, **7:**e41361.

Supplementary figures

Figure S1:

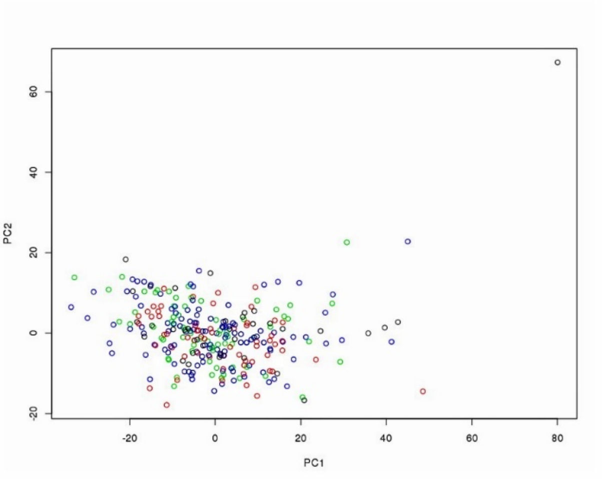

Figure S1: PCA plot of β-methylation for the four sample groups, showing one outlier. The plot is coloured by different groups. Blue group did not develop MetS, but received CBCT, green did not develop MetS and did not receive CBCT, red developed MetS and received CBCT, and black developed MetS and did not receive CBCT.

Figure S2:

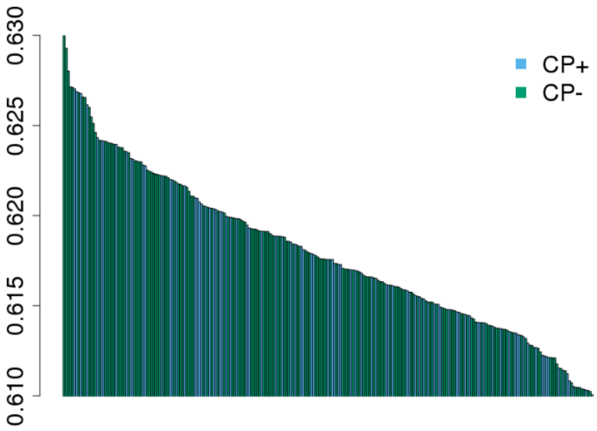

Figure S2. Barplot of the global average methylation per sample. Blue and green indicate whether patients had received cisplatin or not, respectively. Samples were sorted descending using their average methylation value.

Figure S3:

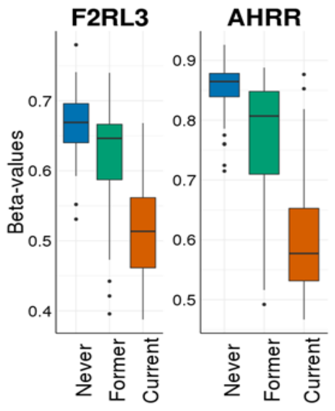

Figure S3. Boxplot of smoking associated CpGs for the genes *AHRR* and *F2RL3*. Never, Former and Current, refer to the smoking status as presented in Table 1.

Fig S4. Q-Q plots for A) CBCT model, methylation β-value as the dependent and CBCT as the independent variable, adjusted for smoking, age, and cell count. B) MetS model, MetS as the dependent and methylation β-value as the independent variable, adjusted for CBCT, smoking, age and cell count.

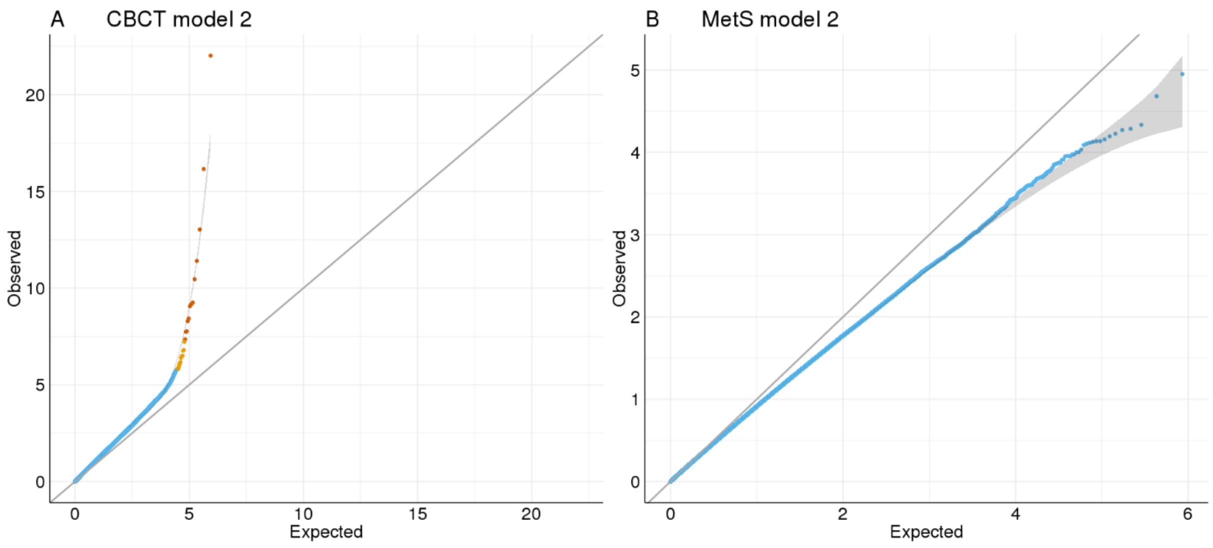

Fig S5. Q-Q plots for A) CBCT model, methylation β-value as the dependent and CBCT as the independent variable, adjusted for smoking, and age. B) MetS model, MetS as the dependent and methylation β-value as the independent variable, adjusted for CBCT, smoking, age, testosterone and cell count.

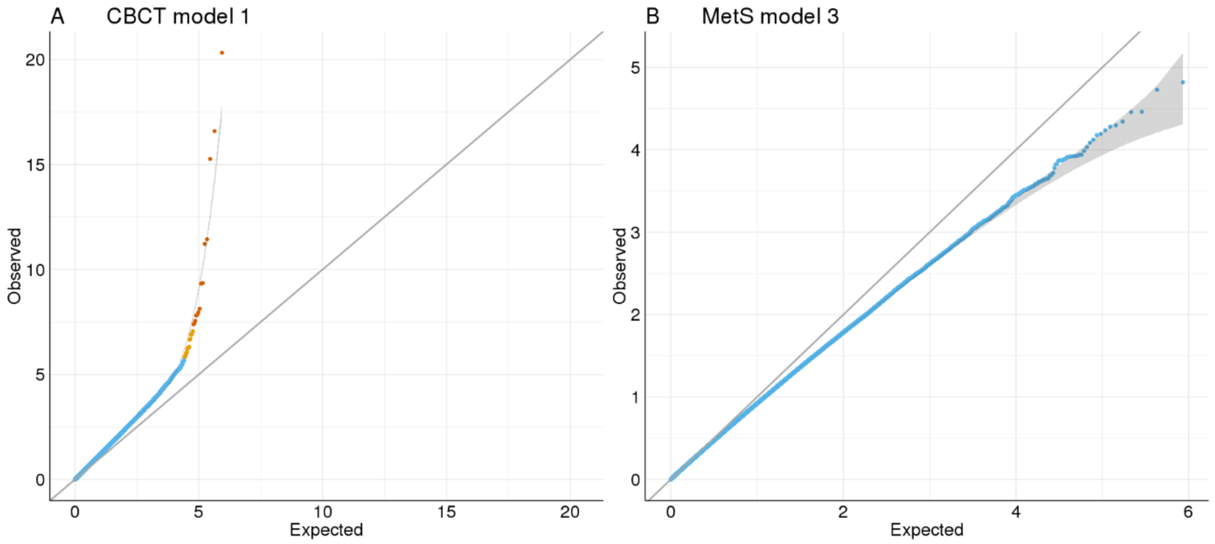

Fig S6. Venn-diagram illustrating the overlapping number of top 2000 nominally significant CpGs between the original model, and the models with the 5 individual criteria of the MetS-diagnosis as dependent variable. Criteria is according to the National Cholesterol Education Program expert panel: Hypertension = blood pressure ≥130/85 mmHg, HDL = HDL-cholesterol <1.0 mmol/L, Triglycerides = triglycerides ≥1.7 mmol/L, Waist Circ. = waist circumference >102 cm, and Glucose = fasting glucose ≥6.5 mmol/L

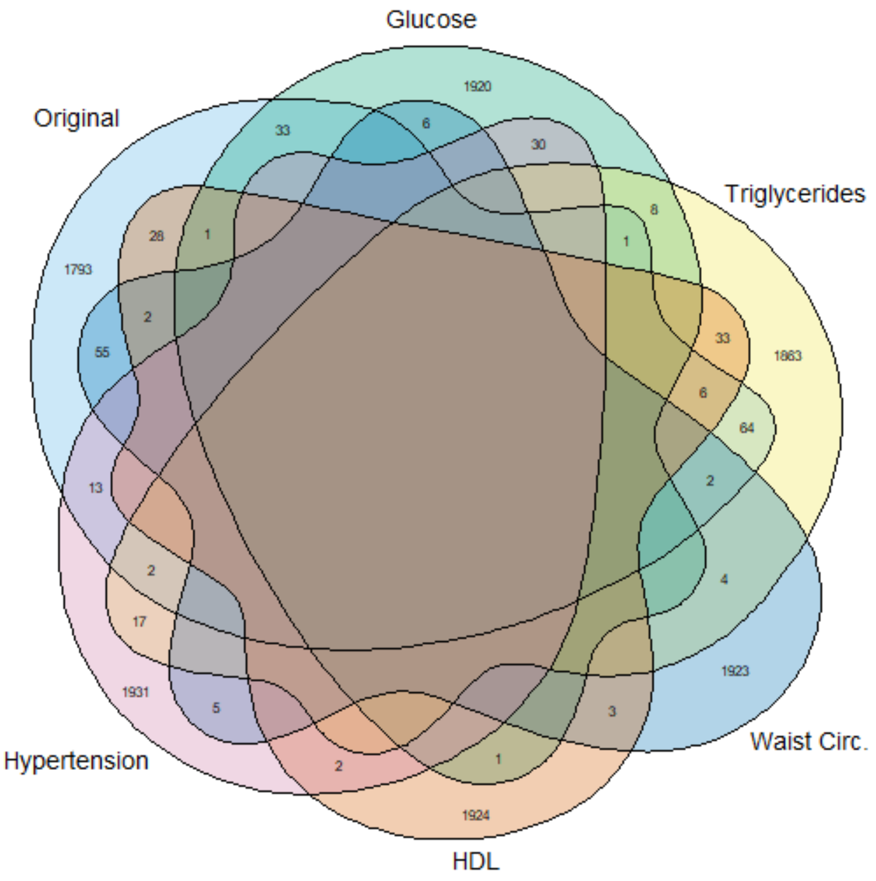

Supplement: Supplementary file 1 — Additional file 1: Figure S1. PCA plot of β-methylation for the four sample groups, showing one outlier. The plot is coloured by different groups. Blue group did not develop MetS, but received CBCT, green did not develop MetS and did not receive CBCT, red developed MetS and received CBCT, and black developed MetS and did not receive CBCT. Figure S2. Barplot of the global average methylation per sample. Blue and green indicate whether patients had received cisplatin or not, respectively. Samples were sorted descending using their average methylation value. Figure S3. Boxplot of smoking associated CpGs for the genes AHRR and F2RL3. Never, Former and Current, refer to the smoking status as presented in Table 1. Figure S4. Q-Q plots for A) CBCT model, methylation β-value as the dependent and CBCT as the independent variable, adjusted for smoking, age, and cell count. B) MetS model, MetS as the dependent and methylation β-value as the independent variable, adjusted for CBCT, smoking, age and cell count. Figure S5. Q-Q plots for A) CBCT model, methylation β-value as the dependent and CBCT as the independent variable, adjusted for smoking, and age. B) MetS model, MetS as the dependent and methylation β-value as the independent variable, adjusted for CBCT, smoking, age, testosterone and cell count. Figure S6. Venn-diagram illustrating the overlapping number of top 2000 nominally significant CpGs between the original model, and the models with the 5 individual criteria of the MetS-diagnosis as dependent variable. Criteria is according to the National Cholesterol Education Program expert panel: Hypertension = blood pressure ≥130/85 mmHg, HDL = HDL-cholesterol <1.0 mmol/L, Triglycerides = triglycerides ≥1.7 mmol/L, Waist Circ. = waist circumference >102 cm, and Glucose = fasting glucose ≥6.5 mmol/L. [file 13148_2019_764_MOESM1_ESM.pdf]
